# Supplementary material for: Realization of large-area ultraflat chiral blue phosphorene
Source: Nat Commun. 2024 Feb 7;15:1157. doi: 10.1038/s41467-024-45263-6 (PMC10850065; doi:10.1038/s41467-024-45263-6)
Supplement: Supplementary file 1 — Supplementary Information [file 41467_2024_45263_MOESM1_ESM.pdf]

Supplementary Information for
Realization of large-area ultraflat chiral blue phosphorene

Ye-Heng Song[1,2,7], M. U. Muzaffar[3,7], Qi Wang[1], Yunhui Wang[1], Yu Jia[1,4,5], Ping Cui[3], Weifeng Zhang[1,2]*, Xue-Sen Wang[6]*, and Zhenyu Zhang[3]*

[1]Center for Topological Functional Materials, and Henan Key Laboratory of Photovoltaic Materials, Henan University, Kaifeng 475004, China

[2]Institute of Quantum Materials and Physics, Henan Academy of Sciences, Zhengzhou 450046, China

[3]International Center for Quantum Design of Functional Materials (ICQD), and Hefei National Laboratory, University of Science and Technology of China, Hefei, Anhui 230026, China

[4]School of Materials Science and Engineering, Henan University, Kaifeng 475004, China

[5]International Laboratory for Quantum Functional Materials of Henan, Zhengzhou University, Zhengzhou 450003, China

[6]Department of Physics, National University of Singapore, 2 Science Drive 3, Singapore, 117542

[7]These authors contributed equally: Ye-Heng Song, M. U. Muzaffar.

*Corresponding author. Email: wfzhang@henu.edu.cn, phywxs@nus.edu.sg, zhangzy@ustc.edu.cn

contents

Supplementary Note 1. Lattice mismatch and moiré period.

Supplementary Note 2. Determination of the period of chiral superstructure and the twisted angle between ultraflat chiral BlueP and Cu(111) substrate.

Supplementary Note 3. Illustration of the geometrical phase analysis (GPA) method

Supplementary Note 4. Stark-shifted image-potential states and local work functions

Supplementary Note 5. The evolution of the phosphorene structures as the coverage varies

Supplementary Note 6. Calculation of charge density distribution of the ultraflat BlueP on Cu(111)

Supplementary Note 7. The atomistic mechanism in the initial growth stages of ultraflat BlueP on Cu(111)

Supplementary Note 1. Lattice mismatch and moiré period.

In reciprocal space, the superlattice wavevector $\mathbf{k}_s = \mathbf{k}_1 - \mathbf{k}_2$, where the subscripts correspond to two different atomic lattices. Here, 1 represents the top compressed Cu layer (matching with the ultraflat chiral BlueP lattice), and 2 represents the bottom uncompressed Cu(111) layer (correspond to bulk Cu). For a hexagonal lattice, the period of the superlattice is given by

$$\lambda_s = \frac{a_1 a_2}{\sqrt{a_1^2 + a_2^2 - 2a_1 a_2 \cos\theta_{12}}}$$

Where θ_{12} is the angle between two different atomic lattices 1 and 2. From the STM results, the a_1 (equal to ultraflat BlueP lattice constant) is ~4.18 Å, a_2 is 4.41 Å [Cu(111)-$\sqrt{3}\times\sqrt{3}$ lattice constant], and the period λ_s of the superlattice is ~4.7nm (close to $11a_1$). Therefore, from the above formula, we can extract the angle of θ_{12} ~ 4.23°. Figure. 3a is the simulation of the moiré pattern where a_1 and a_2 use the experimental value, and twisted angle θ_{12} take the +4.23°. In previous work [1], the twisted graphene bilayer presents chirality due to misaligned stacking breaking the mirror symmetry. The twisted angle θ could be positive or negative, corresponding to left-handed chirality or right-handed chirality. The ultraflat BlueP studied here also presents chirality, showing a near-11×11chiral superstructure. Supplementary Figs. 8a and 8b are the simulated results of moiré patterns where the twisted angle θ take the value of -4.23° and +4.23°, respectively. One can find that these two images are mirror symmetrical. As shown in Supplementary Fig. 8a, the angle between the moiré lattices of left and right-handed chiral regions is ~7.5°, consistent well with the experimentally measured value of ~8°(the angle between two chiral superstructure lattices, see Fig. 2a, further supporting our proposed model. From our observations (Supplementary Fig. 21), the ratio of left and right-handed chiral phases is close to 1:1, consistent with our proposal that the chiral phases are energetically degenerate. Supplementary Figs. 8c and 8d are the zoom-in images of Supplementary Figs. 8a and 8b. Let's focus on the center region; one can find that they show contrary spiral patterns corresponding to different chirality. After the structure relaxed, near-FCC and near-HCP regions transformed into FCC and HCP regions separated by a web-like network of borderlines, which interacted in the nodes. Due to spiral patterns of the unrelaxed structure being contrary, the borderlines

interacted in the nodes also show contrary spiral patterns. Thus, the mirror symmetry of the ultraflat BlueP studied here was broken, then the chirality emerged.

We note that there are bright protrusions at the nodes. The atomic resolution STM image (see Supplementary Fig. 13) shows that these bright protrusions are composed of three P atoms (a P trimer). From the line-scan profile across the protrusions (see Supplementary Fig. 4), the measured height of the protrusion is ~0.8 Å, indicating that the phosphorous trimers are located on top of the phosphorene layer. Most of the bright protrusions are phosphorous trimers, and only a small portion of them are dimers or tetramers (see Fig. 2). As proposed in the schematic diagrams of Figs. 3a and 3b, at the node, the first-layer atom is located at the on-top site, which is not a stable site. Therefore, the atoms in the nodal area relax via atomic displacement to minimize the total energy. As shown in Supplementary Fig. 13c, there exist obvious misfit dislocations in the nodal area due to the atomic displacement, as guided by the black lines. Therefore, this chiral pattern is very different from the moiré spots as reported in the buckled BlueP[2] grown on the Cu(111).

Supplementary Note 2. Determination of the period of chiral superstructure and the twist angle between ultraflat chiral BlueP and Cu(111) substrate.

In the main text, we have discussed the formation process of the chiral superstructure, in which the relaxed structure (chiral superstructure) evolves from the moiré superstructure. Therefore, the period of the chiral superstructure should be relative to the moiré superstructure. For the chiral superstructure of ultraflat BlueP studied here, its period equates to the distance of two nodes. Here, we measured the period of the chiral superstructure in the reciprocal space. Supplementary Fig. 5a is the high resolution STM image of the chiral superstructure, the corresponding FFT image is shown in Supplementary Fig. 5b, where the yellow circles mark the Bragg peaks of the lattice and the green circles mark the Bragg peaks of the chiral superstructure. For more reliability, we made statistics on more than 20 areas to obtain the ratio between the ultraflat BlueP lattice and chiral superstructure's period. The statistical results are displayed in Supplementary Fig. 5c, in which the mean value of the period is $\sqrt{124.9}a$ (a is the lattice of ultraflat BlueP), ~4.7nm, close to $11a$.

Supplementary Note 3. Illustration of the geometrical phase analysis (GPA) method

The basic idea of GPA is as follows: If the sample has a certain strain (it must be noted that the micro strain), its lattice constant should have a certain change, and the lattice may be enlarged, shrunk, or distorted. Depending on certain geometric operations, the size of the strain can be qualitatively or even quantitatively judged from the atomic-resolution TEM or STM images. The theoretical basis of relevant contents was published in *Ultramicroscopy* in 1998. The geometrical phase analyze method was first used to analysis the strain of lattice distortion in HRTEM image by R. Kilaas et al.[3]. Here, we also use this method to analyze the lattice distortion of the ultraflat BlueP induced by interface strain via high resolution STM image. Here, we briefly describe how it works:

1. To obtain the frequency spectrum image by performed Fourier transform on the high resolution STM image.

2. To perform center filtering around the diffraction points.

3. Choosing a Bragg peak, and then perform the inverse Fourier transform

4. Calculating the geometric phase of the inverse Fourier transform image.

5. Calculating the local lattice distortion, a gradient matrix of displacement field.

The detail of the mathematical process of this method is as follows:

The atomic-resolution STM image can be written as $T(r) = A_0 + \sum_g 2A_{\boldsymbol{g}}(\boldsymbol{r}) \cos\{2\pi\boldsymbol{g}\cdot\boldsymbol{r} + P_g(\boldsymbol{r})\}$ after the real function Fourier transform, where g is the reciprocal lattice vector describing the undeformed lattice, $A_{\boldsymbol{g}}(\boldsymbol{r})$ is the amplitude and $P_{\boldsymbol{g}}$ is the phase.

After Fourier transform, place a mask around the ±g diffraction spots. Then perform inverse Fourier transform to obtain a complex image corresponding to a specific spatial frequency g:

$$I'_g(r) = A_g(\boldsymbol{r})\exp\{i\cdot[2\pi\boldsymbol{g}\cdot\boldsymbol{r} + P_g(\boldsymbol{r})]\}$$

Based on the above formula, the image intensity, amplitude image, and geometric phase image of the Bragg filter can be calculated. The parameter we are most concerned with is the geometric phase,

$$P_g(\boldsymbol{r}) = Phase[I'_g(r)] - 2\pi \boldsymbol{g} \cdot \boldsymbol{r}$$

As the core equation of GPA method is $P_g(\boldsymbol{r}) = -2\pi \boldsymbol{g} \cdot \boldsymbol{u}$. From this relation, as long as the phase image $P_g(\boldsymbol{r})$ is obtained, the displacement field $\boldsymbol{u}$ can be obtained. This result is the core equation of GPA analysis method. Next, select two non-linear correlated reciprocal lattice vector diffraction g_1 and g_2 in the Fourier transform image, and use the core equation to get the following results:

$$P_{g1}(\boldsymbol{r}) = -2\pi \boldsymbol{g_1} \cdot \boldsymbol{u} = -2\pi\{g_{1x}u_x(r) + g_{1y}u_y(r)\}$$

$$P_{g2}(\boldsymbol{r}) = -2\pi \boldsymbol{g_2} \cdot \boldsymbol{u} = -2\pi\{g_{2x}u_x(r) + g_{2x}u_y(r)\}$$

After measuring the phase images, the displacement field u can be determined as

$$\begin{pmatrix} u_x \\ u_y \end{pmatrix} = -\frac{1}{2\pi}\begin{pmatrix} g_{1x} & g_{1y} \\ g_{2x} & g_{2y} \end{pmatrix}^{-1}\begin{pmatrix} P_{g_1} \\ P_{g_2} \end{pmatrix}$$

The local distortion of the lattice $\boldsymbol{e}$ is given by the gradient of the displacement field $\boldsymbol{u}$:

$$e = \begin{pmatrix} e_{xx} & e_{xy} \\ e_{yx} & e_{yy} \end{pmatrix} = \begin{pmatrix} \frac{\delta u_x}{\delta x} & \frac{\delta u_x}{\delta y} \\ \frac{\delta u_y}{\delta x} & \frac{\delta u_y}{\partial y} \end{pmatrix}$$

From the strain tensor matrix $\varepsilon = \frac{1}{2}\{e + e^T\}$, the plane strain can be written as:

$$\begin{cases} \varepsilon_{xx} = e_{xx} \\ \varepsilon_{yy} = e_{yy} \\ \varepsilon_{xy} = \varepsilon_{yx} = \frac{1}{2}(e_{xy} + e_{yx}) \end{cases}$$

Supplementary Note 4. Stark-shifted image-potential states and local work function

In STM, when the bias voltage of the sample over the local work function, the electrons tunnel in the Fowler−Nordheim regime. The high electric filed in the tunnel junction results in Stark-shifted image potential states (as illustrated in Fig. 4c). From a quasi-classical approximation, when the sample bias voltages align with one of these shifted image potential states, resonant tunneling occurs[4, 5].

$$eV_n = \phi + \left(\frac{3n\pi\hbar eE}{2\sqrt{2m}}\right)^{2/3} \quad (1)$$

where V_n is the sample voltage of nth resonant, ϕ is the local work function, and E is the electric filed.

Supplementary Note 5. The evolution of the phosphorene structures as the coverage varies

As the focused phosphorene structure in our experiments, the ultraflat chiral BlueP cannot be directly obtained on Cu(111) but is obtained from the transition of other phosphorene structure as the P coverage increases. In our experiment, we observed four different phosphorene structural phases, including the chain, strip, chiral, and hexagonal phases, as shown in Fig. 5. We found that the phosphene structural phases grown on Cu(111) depends critically on the phosphorous coverage (here, 1ML phosphorene is defined as the surface is fully covered by ultraflat chiral BlueP) at a proper substrate temperature (200-250°C). First, when the coverage is below about ~0.85 ML, a chain phosphorene structure is obtained. Supplementary Fig. 23a shows the STM topographic image with a coverage of ~0.5 ML, showing that a chain structure and Cu(111) surface coexist. When the coverage is up to ~0.85 ML, the Cu(111) surface is fully covered by the chain phase (see Supplementary Fig. 23b). Further depositing phosphorus (> 0.85 ML), the chain structure phase will transform into a strip phase, as shown in Supplementary Fig. 23c, where the chain and strip phases coexist. As the coverage further increases to ~0.95 ML, the chain phase will totally transform into the strip phase (see Supplementary Fig. 23d). If we continue to increase the coverage (> 0.95 ML), the strip phase starts to transform into the chiral phase. Supplementary Fig. 23e shows the STM image after the deposition of ~0.97 ML phosphorus, where the strip and chiral phase coexist. When the

coverage is up to 1 ML, the chiral phase would fully cover the Cu(111) surface (see Supplementary Fig. 23f). As we continue to deposit phosphorous (>1 ML), the chiral phase slowly converts to the hexagonal phase (see Supplementary Fig. 23g and Fig. 38a). In the end, the subsequently-arrived P atoms are adsorbed on the nodes, forming self-assembled P nanodots (as shown in Supplementary Fig. 23h and Fig. 38b; we also name it hexagonal phase as it holds a hexagonal superstructure as well) located on the top of the phosphorene layer. As shown in Supplementary Fig. 24, the apparent height of the P nanodots is ~2 Å, indicating that the P nanodots are located on the top of the phosphorene layer. The measured period of the P nanodots is ~ 3.1 nm. The observed apparent height and period of the P nanodots are consistent with the work[2] of Kaddar et al., suggesting that the self-assembled P nanodots are of the same structure on the ultraflat (here) and buckled BlueP.

Interestingly, we can also controllably decrease the P coverage through annealing. Under proper annealing temperature (~350°C), the P atoms can slowly desorb from the phosphorene sheet, resulting in reversed phase transitions (see Fig. 5). These experimental results indicate that the formation of all the phosphorene structures on Cu(111) sensitively depends on the coverage at a proper substrate temperature (200-250°C). Through precise control of the coverage, one can select a single phosphorene phase on the Cu(111) surface. As shown in Supplementary Fig. 19, the large-area ultraflat chiral BlueP fully covers the Cu(111) substrate surface, which is only limited by the size of our actual STM probe. When the substrate temperature is below 200°C, such as 180 °C, multiple phases will coexist on the surface (see Supplementary Fig. 25), including the chain, strip, and chiral phases. We suggest that, at the low surface temperature, the P atoms or dimers can migrate relatively short distances on the surface, resulting in different local coverages (local surface atomic densities of P atoms) of various areas. Therefore, several phosphorene structures could appear on the surface even when the coverage is below 1ML.

For the chiral phase, an ultraflat honeycomb structure similar to graphene is demonstrated by the high-resolution STM image and DFT calculations (see Fig. 1). According to the high-resolution STM images of phosphorene structures (see Fig. 5e-h), we mark the unit cells of different phosphorene structures. One can see that the chiral and hexagonal phases hold a

similar hexagonal unit cell. The strip phase has a pseudo-hexagonal unit cell (oblique unit cell) that is very close to the chiral phase. The unit cell of the chain phase shows a large difference from the other three phases at first glance. However, when carefully comparing it with the strip phase, one can find that it is close to the 2×1 unit cell of the strip phase. Based on these results, we propose the atomic structures of the other three phosphorene phases. As shown in Supplementary Fig. 40, all the phosphorene structures show ultraflat honeycomb or pseudo-honeycomb structures. Specifically, the chiral and hexagonal phases have an ultraflat honeycomb structure similar to graphene; the strip phase shows a pseudo-honeycomb structure that is close to the chiral phase; and the chain phase can be regarded as a further deformation of the pseudo-honeycomb structure of the strip phase. Based on these proposed atomic structures, one can obtain the surface atomic densities for different phosphorene phases and then obtain the coverages of the different phosphorene phases that fully cover the Cu(111) surface. Table S1 displays the coverages of different phosphorene phases estimated from the proposed atomic structures and obtained from the experiments. One can see that the coverages calculated by proposed structures are consistent with the experimental results, further supporting our proposed models. Since these phosphorene phases have similar atomic structures (see Supplementary Fig. 40), they can easily undergo structural evolution as the coverage increases, or reverse as the coverage decreases (see Fig. 5).

The proposed atomic structure of the chiral phase is well consistent with the high-resolution STM image (see Fig. 1g), which shows an apparent ultraflat honeycomb structure akin to graphene. For the other three phosphorene phases (including chain, strip, and hexagonal phases), the high-resolution STM images cannot distinguish all the P atoms but only show bright protrusions in hollow sites (see Fig. 5e, f, h). This phenomenon has been reported in previous works of Borophene on Ag(111)[4, 6], in which the high-resolution STM images cannot distinguish the B atoms due to the influence of the local electronic states (LDOS). In STM, the tunneling current depends strongly on the LDOS of a surface; therefore, the measured height of a local area may not represent the actual height but the LDOS strength. To fully determine the atomic structure of these phosphorene phases, atomic-resolution images are needed. The q-plus AFM can provide sub-atomic resolution images for surface structures without the influence

from the LDOS. It should be desirable to reveal the structural evolution of the phosphene phase as revealed in detail by high-resolution q-plus AFM and corresponding DFT calculations in a future study.

In addition, we noted that the recent work[2] of Kaddar et al. reported the fabrication of a buckled BlueP with a lattice period of 3.4 Å on Cu(111). In their work, they used a higher growth temperature (~260 °C) and black phosphorus as the precursor. As a comparison, in our experiments, we use a relatively low growth temperature (200-250°C) and InP as the precursor. To investigate why BlueP has such a tremendous difference on the same substrate, we have tried to grow phosphorene at a higher growth temperature. Supplementary Fig. 22 shows the STM topographic images of the phosphorene structures grown at ~260 °C (the growth temperature used in the work[2] of Kaddar et al.) and still uses InP as the precursor, which also shows the same phosphorene structures (chain, strip, and chiral phases) as the results of Fig. 5 (substrate temperature: 200-250°C). It is known that at temperature below 800 °C, when using black phosphorus as the precursor, phosphorus vapor mainly consists of P_4 molecules[7]. However, with InP in the evaporator, the phosphorus vapor mainly consists of P_2 molecules[8]. Therefore, we speculate that the different phosphorus precursors might result in different phosphorene structures on Cu(111). However, we cannot rule out the influence of the growth temperature. Due to certain deviations between the measured substrate temperature and the actual substrate temperature, which is different for different MBE systems, the actual temperature used in our experiment might not be the same as the work[2] of Kaddar et al. Therefore, to further understand the influence of the phosphorus precursor and growth temperature on phosphorene structures grown on Cu(111), more control and comparative experiments are needed.

Supplementary Note 6. Calculation of charge density distribution of the ultraflat BlueP on Cu (111)

To quantify the amount of charge transfer, we have calculated the charge density distribution of the ultraflat BlueP on Cu (111), as shown in Supplementary Fig. 35. The charge density difference $\Delta\rho c$ is defined as the plane-averaged charge difference along the c-axis between the

BlueP/Cu(111) system and the sum of the isolated BlueP monolayer and Cu(111) substrate. It can be seen that when the BlueP and Cu layers merge into the BlueP/Cu(111), an electron-depletion region is formed near the top most layer of Cu, and this amount of electron charge is transferred to the interfacial region. Taking a physically intuitive reference plane where the total charge is a minimum (highlighted by the black dash line in Supplementary Fig. 35b), the amount of electron transfer can be obtained by integrating Δρc over the c-axis within the BlueP region. The net charge transfer is calculated to be 0.018 e/f.u. from the Cu(111) substrate to BlueP. For comparison, we have also calculated the charge transfer through the Bader charge analysis. Our calculated results reveal that the charge transfer from the Bader charge analysis (0.09 e/f.u.) and through integrating the charge density difference distribution (0.018 e/f.u.) show the same direction of charge transfer, but the former approach could overestimate its magnitude because the Bader analysis relies on the value of peaked charge. Such a significant charge transfer and a strong binding energy (0.56 eV/P) explain the strong interaction between the P atoms and Cu(111), resulting in the formation of ultraflat BlueP.

Supplementary Note 7. The atomistic mechanisms in the initial growth stages of ultraflat BlueP on Cu(111).

It is well-established that the initial stages of growth, characterized by the atomistic processes of adsorption, diffusion, nucleation, and islanding, play decisive roles in dictating the dominant growth mode(s) eventually selected[9]. In this regard, we have explored the adsorption and diffusion properties of P adatom(s) on Cu(111), and the results are depicted in Supplementary Figs. 36 and 37. Our detailed calculations reveal that a single P adatom prefers to occupy valley sites, as shown in Supplementary Fig. 36a. When two P atoms are placed on the surface, they prefer to form a dimer with a P-P distance of 2.11 Å rather than staying apart (see Supplementary Fig. 36). The calculated adsorption energies are -6.05 eV/P and -6.12 eV/P, respectively, for P monomer and dimer on Cu(111). The adsorption energy is defined as $E_{ads} = E_{total} - nE_P - E_{sub}$, where E_{total}, E_P, and E_{sub} are the total energies of the combined system, an isolated P atom in gas phase, and the Cu(111) substrate, respectively and n corresponds to number of P adatom(s). Furthermore, we have investigated the adsorption of an isolated P_2 and P_4 on Cu(111), aiming to understand the role of the precursor in the nucleation and epitaxial

growth of BlueP. The corresponding adsorption energy is defined as $E_{ads} = (E_{total} - E_{P2/P4} - E_{sub})$, where E_{total} and E_{sub} are the total energies of the combined system and pristine Cu(111) substrate, respectively, and $E_{P2/P4}$ corresponds to the total energy of an isolated P_2 or P_4 in the gas phase. The resulting E_{ads} are –1.67 eV/P and –0.99 eV/P, respectively, ensuring the stability of both P_2 and P_4 on the substrate.

Next, we have calculated the diffusion properties of both P_2 and P_4 in comparison of P monomer on the Cu(111) substrate. In this regard, we explore various diffusion pathways for migration, as illustrated in Supplementary Fig. 37. Our calculations reveal that the maximum diffusion barrier for a P monomer is 75 meV. A subtle and crucial aspect is that, considering the clear tendency of two P monomers to form a stable dimer, it is also possible that such a P-P dimer may diffuse faster as an entity than the separated monomers. Our detailed calculations indeed confirm this conjecture. As illustrated in Supplementary Fig. 37, the minimum diffusion barrier for the P dimer is significantly reduced to 41.4 meV, even lower than that of P_4. The underlying reason of a stable and fast diffusing dimer on Cu(111) is inherently rooted in the relative binding strengths of P-P and P-substrate[10]. Here, it is also worthwhile to mention that similar dimer feeding behaviors have been proposed for the initial growth stages of graphene on Cu substrates[11], resulting in the growth of large-area and high-quality samples as observed[12, 13]. More importantly, for a P_4 cluster, the diffusion barrier is much higher, at ~0.54 eV (Supplementary Fig. 37). Therefore, we suspect that, in the work[2] of Kaddar et al., the P_4 precursors have to be either first decomposed into P_2 dimers, then fast feed to the growing islands, or have to be supplied at a much slower rate as P_4 entities[14, 15] for chain feeding in the case of graphene). Based on these calculations, we may conclude that two P atoms prefer to form a stable dimer on Cu(111) or P_2 dimers can be directly deposited on Cu(111), and such dimers are energetically stable and can diffuse much faster than either a P monomer or (especially) a P_4 cluster. Thus, the growth precursor (P_2 in the present case) in epitaxial growth may play a decisive role in the formation of ultraflat BlueP on Cu(111), as presented in the present study.

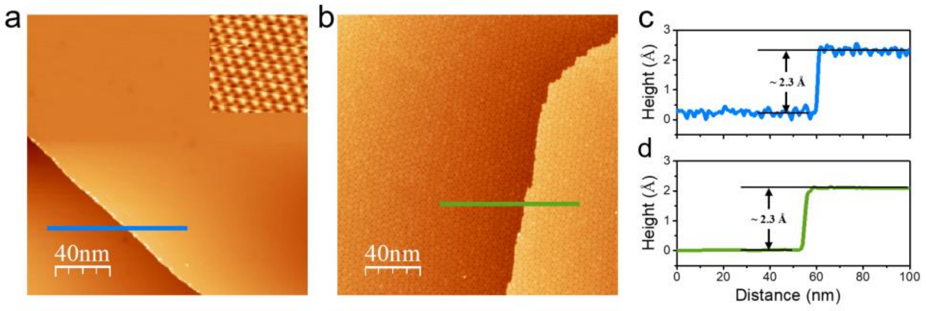

Supplementary Fig. 1 a,**b**, STM topographic images (200×200 nm^2, 78K) of Cu(111) surface and ultraflat chiral BlueP. The inset in **a** is the atomic resolution image (2×2 nm^2, 78K) of Cu(111) surface. **c**,**d**, Corresponding line scan profiles taken across the blue and green lines as guided in **(a-b)**, which all show the same step height of ~2.3 Å.

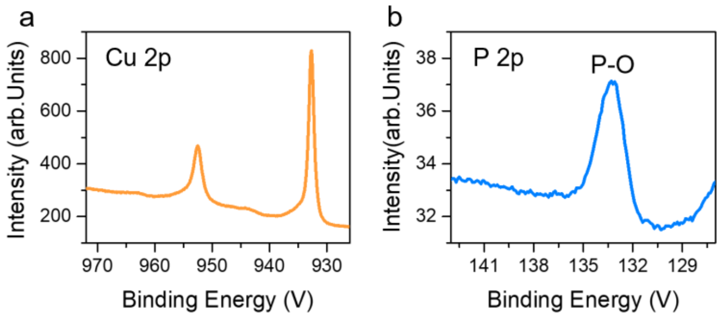

Supplementary Fig. 2 a, XPS spectra of Cu 2p core levels after growth of ultraflat chiral BlueP. **b**, P 2p core-level spectrum, presenting only the P-O peak but not the pure P peak, indicating the ultraflat chiral BlueP is vulnerable to environmental degradation.

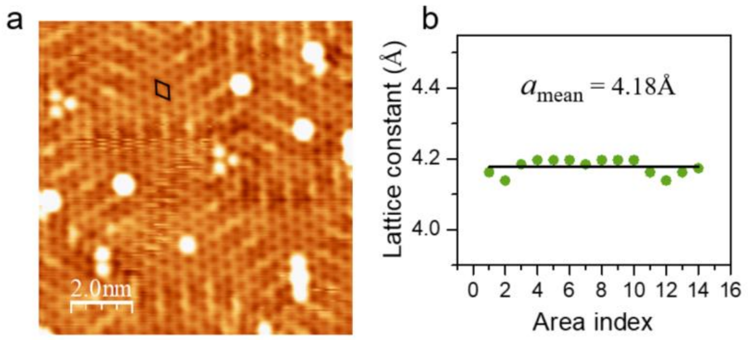

Supplementary Fig. 3 a, Hight resolution STM image (10×10 nm^2, 78K) of ultraflat chiral BlueP. The black diamond marks the unit cell of it. **b**, Statistical average results of the lattice constant of ultraflat chiral BlueP, consistent well with the RHEED result of Fig. 1c.

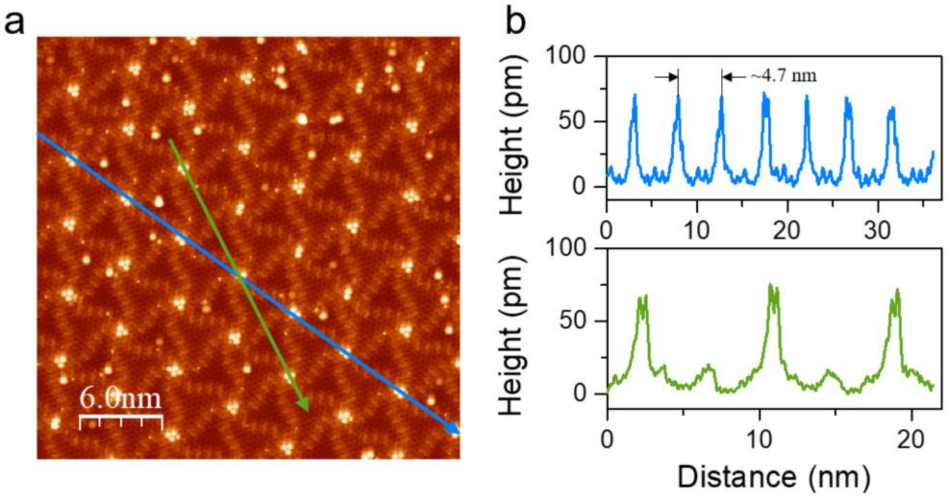

Supplementary Fig. 4 a, Hight resolution STM image (30×30 nm^2) of ultraflat chiral BlueP. **b**, Line-scan profiles along the blue line (top image) and green line (bottom image) as guided in **a**, showing a period of ~ 4.7 nm for chiral superstructure and an apparent height of ~70pm for nodes.

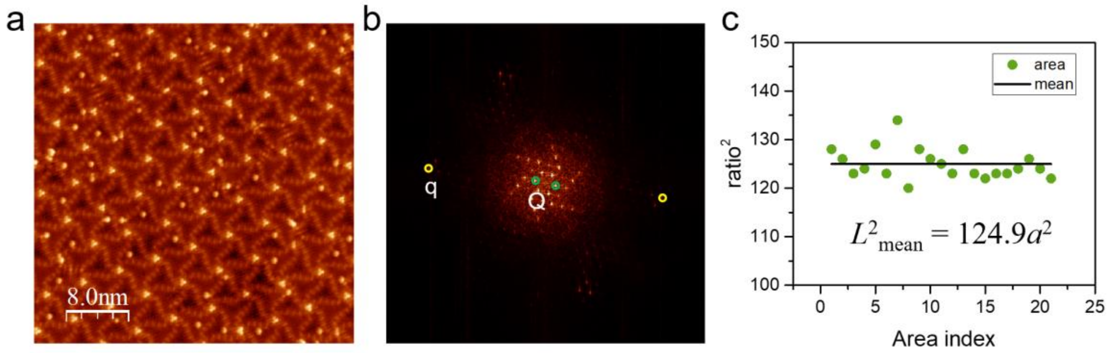

Supplementary Fig. 5 a, Hight resolution STM image (V_S = 2 mV, I_t = 1000 pA) of ultraflat chiral BlueP. **b**, Corresponding Fast Fourier transform of a, the yellow and green circles mark the Bragg peaks of ultraflat BlueP and chiral superstructure, respectively. **c**, Statistical average results of the lattice constant of chiral superstructure.

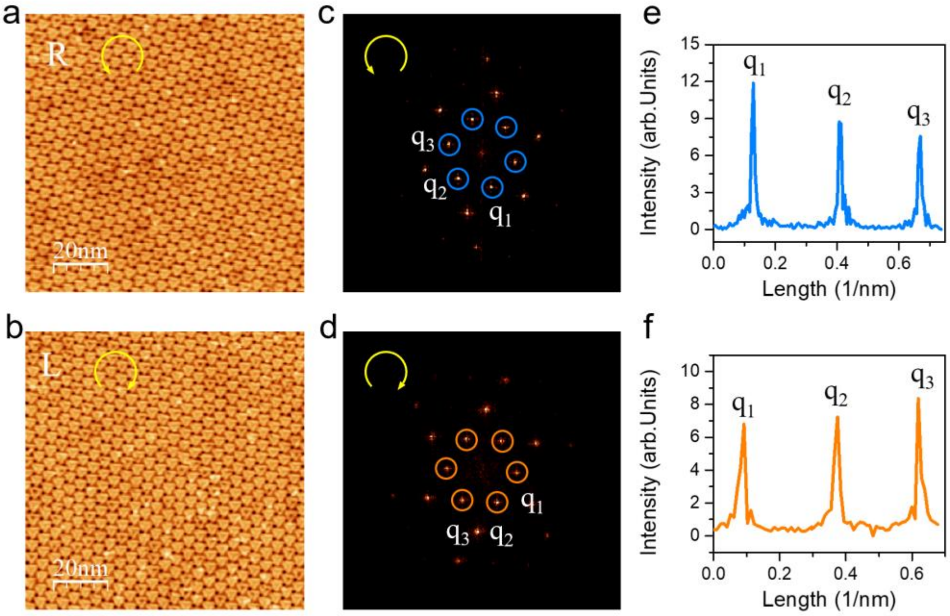

Supplementary Fig. 6 **a**,**b**, Large area STM topographic images (100×100 nm^2) of D1(right-hand chirality) and D2 (left-hand chirality) areas. **c**,**d**, Corresponding Fast Fourier transforms of **(a**,**b)**. The blue and orange circles mark the Bragg peaks of the chiral superstructure. **e**,**f**, Line profiles across the q_1, q_2 and q_3 Bragg peaks as marked in **(c**,**d)**.

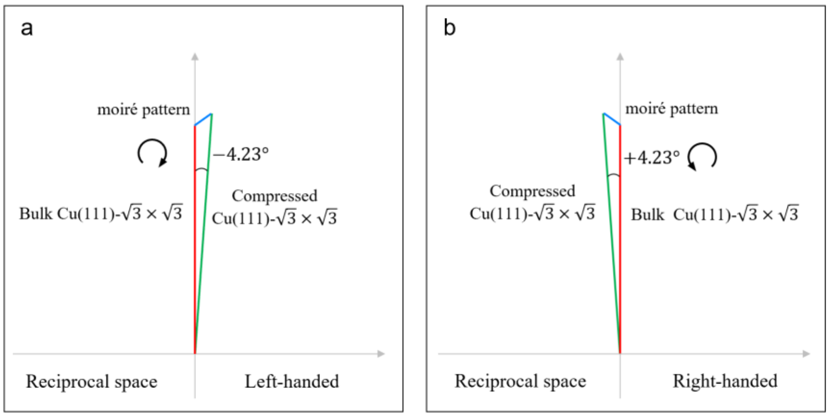

Supplementary Fig. 7 Schematic illustration of the relationship between the wavevectors of ultraflat chiral BlueP, Cu(111)-$\sqrt{3} \times \sqrt{3}$, and moiré period in the reciprocal space. The negative and positive rotational angle θ in **(a**,**b)** correspond to left-handed and right-handed chirality of ultraflat BlueP on Cu(111).

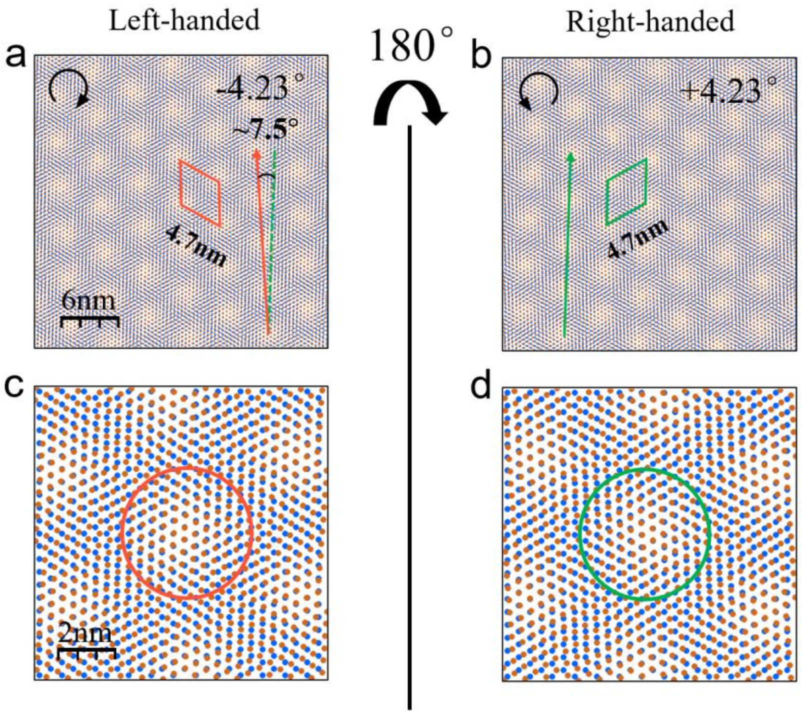

Supplementary Fig. 8 a,**b**, Simple model of placing a layer of compressed Cu (brown dots, matching with the ultraflat chiral BlueP lattice) on a Cu slab (blue dots, not compressed) with a small rotational angle, showing a moiré pattern induced by lattice mismatch. For left model, the twisted angel θ is -4.3°, for right model, the twisted angel θ is +4.3°. Here, in both models, the lattice constants of top layer and bottom layer films are 4.18 Å and 4.41 Å, respectively. **c**,**d**, Zoom-in images corresponding to the center of **(a**,**b)**. When focus on the center of the nodes, one can find the spiral pattern of the nodes is contrary, which is the origin of left-handed and right-handed domains.

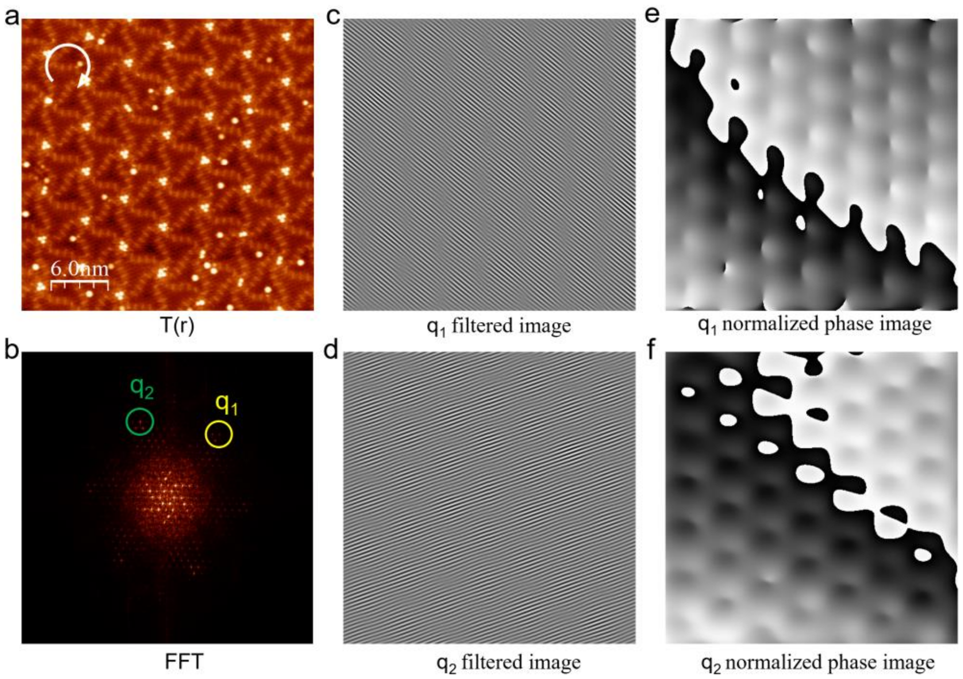

Supplementary Fig. 9 **a**, STM topographic image (30×30 nm^2) of ultraflat chiral BlueP in D2 area. **b**, Corresponding Fast Fourier transforms of **(a)**. The yellow and green circles. Reference lattice $q_1 = a\ [10\bar{1}]$ (red circle) and $q_2 = a\ [01\bar{1}]$ (blue circle) are marked. **c**,**d**, $[10\bar{1}]$ Bragg spots filtered image and normalized phase image. **d**,**f**, $[01\bar{1}]$ Bragg spots filtered image and normalized phase image.

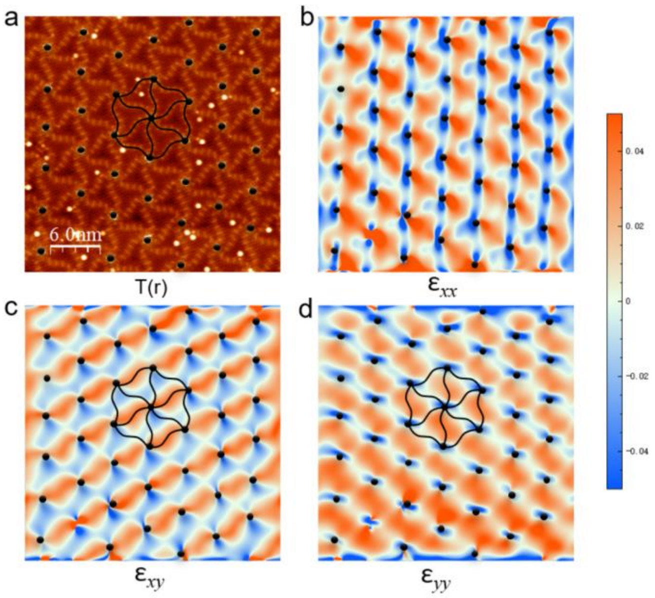

Supplementary Fig. 10 a, High resolution STM image (30×30 nm^2) in right-handed chiral region. **b-d** The ε_{xx}, ε_{xy} and ε_{yy} strain maps of the STM image in **a** generated by the geometric phase analysis method. Here, ε_{xx}, and ε_{yy} are normal strains, ε_{xy} is shear strains. The black dots in **(a-d)** mark the position of the nodes.

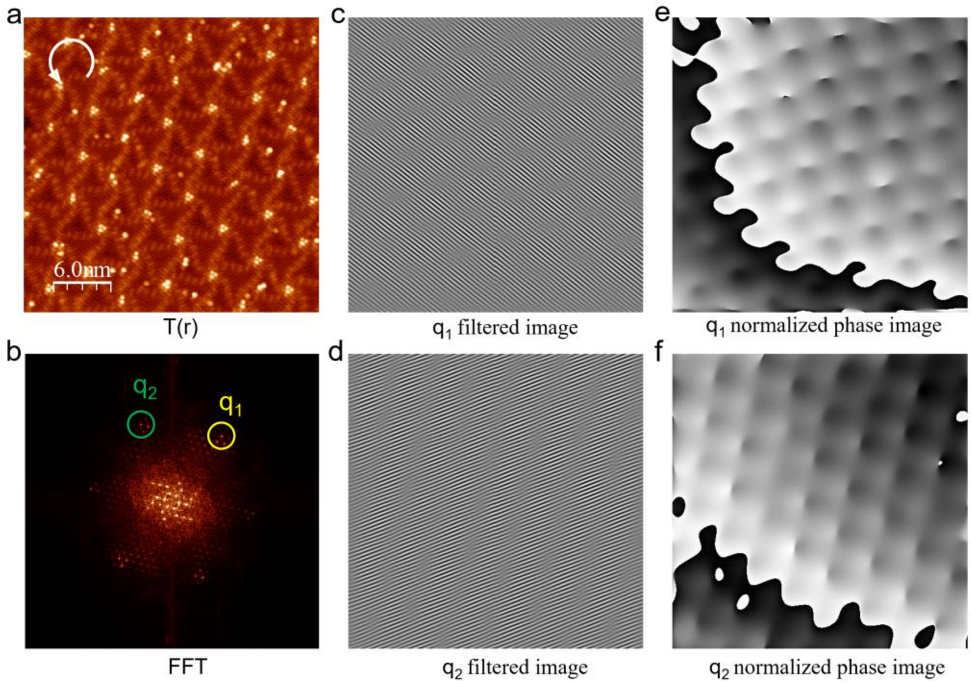

Supplementary Fig. 11 a, STM topographic image (30×30 nm^2) of ultraflat chiral BlueP in D1 region. **b**, Corresponding Fast Fourier transforms of **(a)**. The yellow and green circles. Reference lattice q_1 = a$[10\bar{1}]$ (red circle) and q_2 = a$[01\bar{1}]$ (blue circle) are marked. **c**,**d**, $[10\bar{1}]$Bragg spots filtered image and normalized phase image. **d**,**f**, $[01\bar{1}]$ Bragg spots filtered image and normalized phase image.

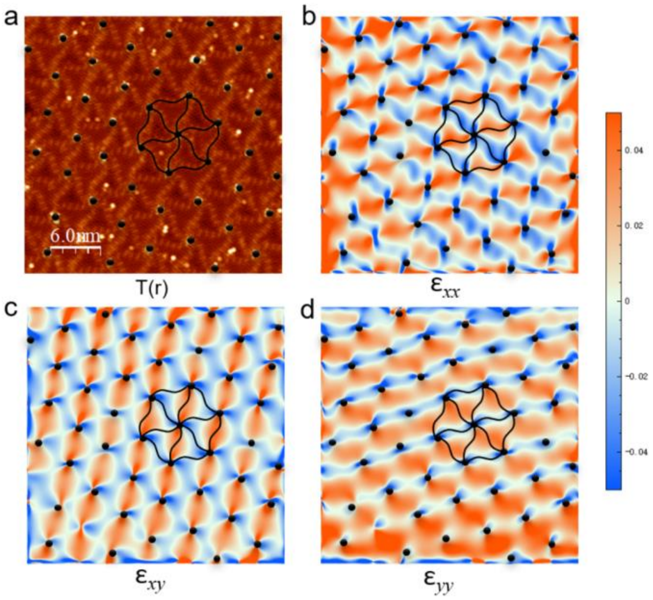

Supplementary Fig. 12 a, High resolution STM image (30×30 nm^2) in left-handed chiral region. **b-d** The ε_{xx}, ε_{xy} and ε_{yy} strain maps of the STM image in **a** generated by the geometric phase analysis method. Here, ε_{xx}, and ε_{yy} are normal strains, ε_{xy} is shear strains. The black dots in **(a-d)** mark the position of the nodes.

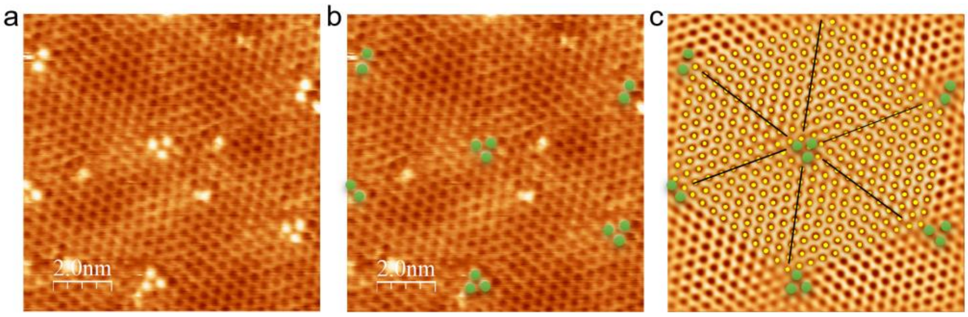

Supplementary Fig. 13 a,**b**, Atomic resolution STM images (10×10 nm^2, 78K) of ultraflat chiral BlueP. The green dots in **(b)** mark the P atoms at the nodes. **b**, Bragg-peaks filtered image corresponding to **(a)**, which can enhance the clarity of the lattice. The green dots mark the P atoms at the nodes, and the yellow dots mark the positions of ultraflat chiral BlueP lattices. There exist obvious misfit dislocations as guided by the black lines.

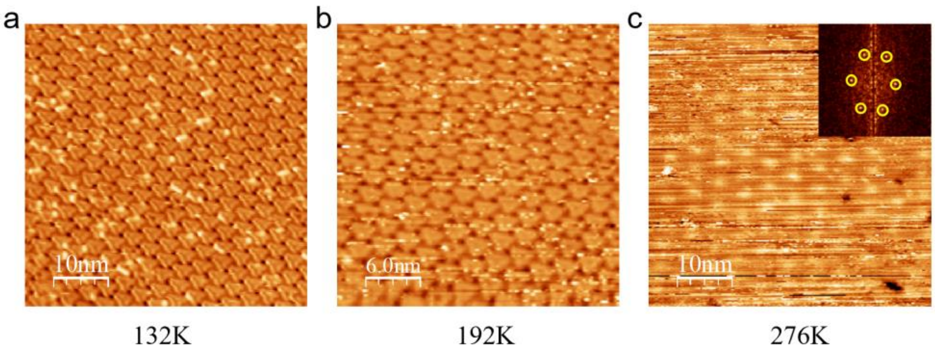

Supplementary Fig. 14 STM topographic images of ultraflat chiral BlueP performed at different temperatures, in which the chiral superstructure always exists.

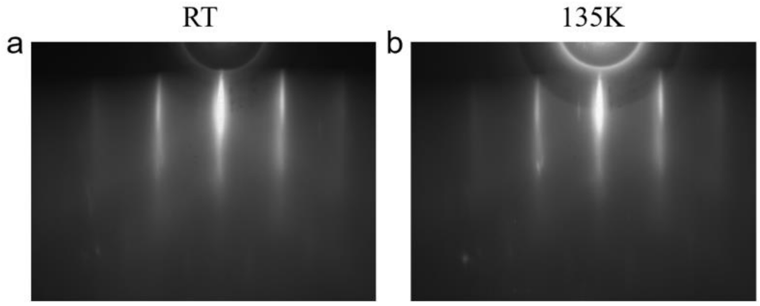

Supplementary Fig. 15 RHEED patterns of ultraflat chiral BlueP on Cu(111) recorded at room temperature and low temperature (135K), which both show the same characteristic.

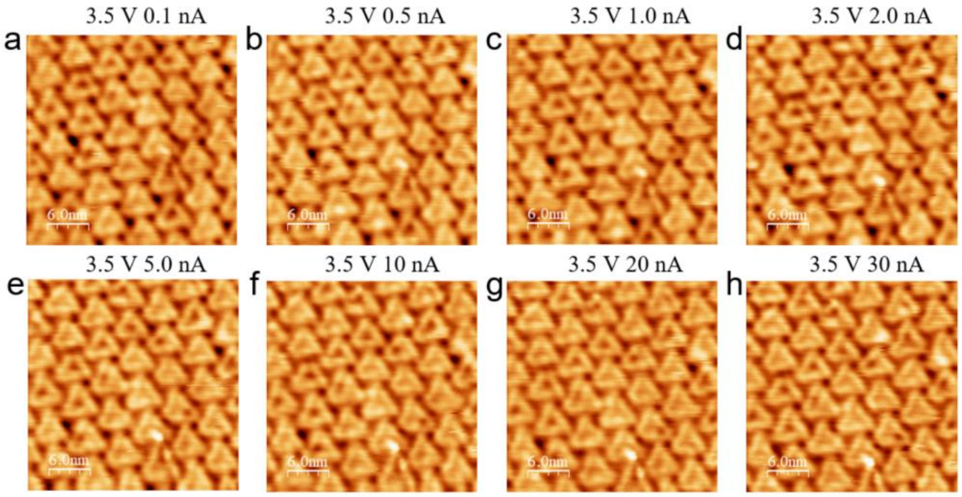

Supplementary Fig. 16 Series of STM topographic images (30×30 nm^2, 78K) of ultraflat chiral BlueP obtained at different tunneling gap.

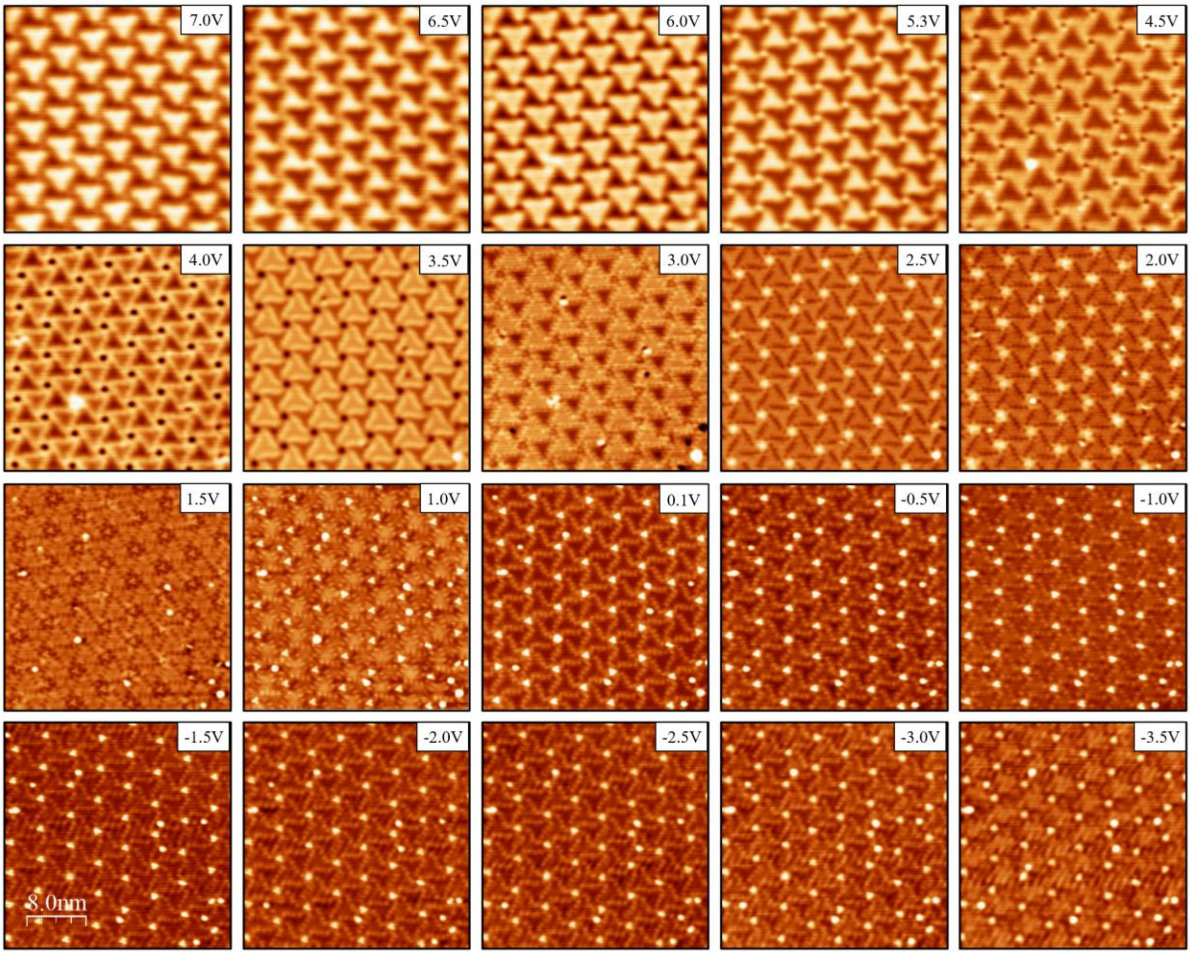

Supplementary Fig. 17 STM morphologies of ultraflat chiral BlueP on Cu(111) with vary bias voltage. Bellow 1.0V, the morphologies show small difference. However, above 1.0 V, the morphologies show rich forms. Specially at high scanning bias, the brightness (apparent height) of triangle units shows oscillatory variation.

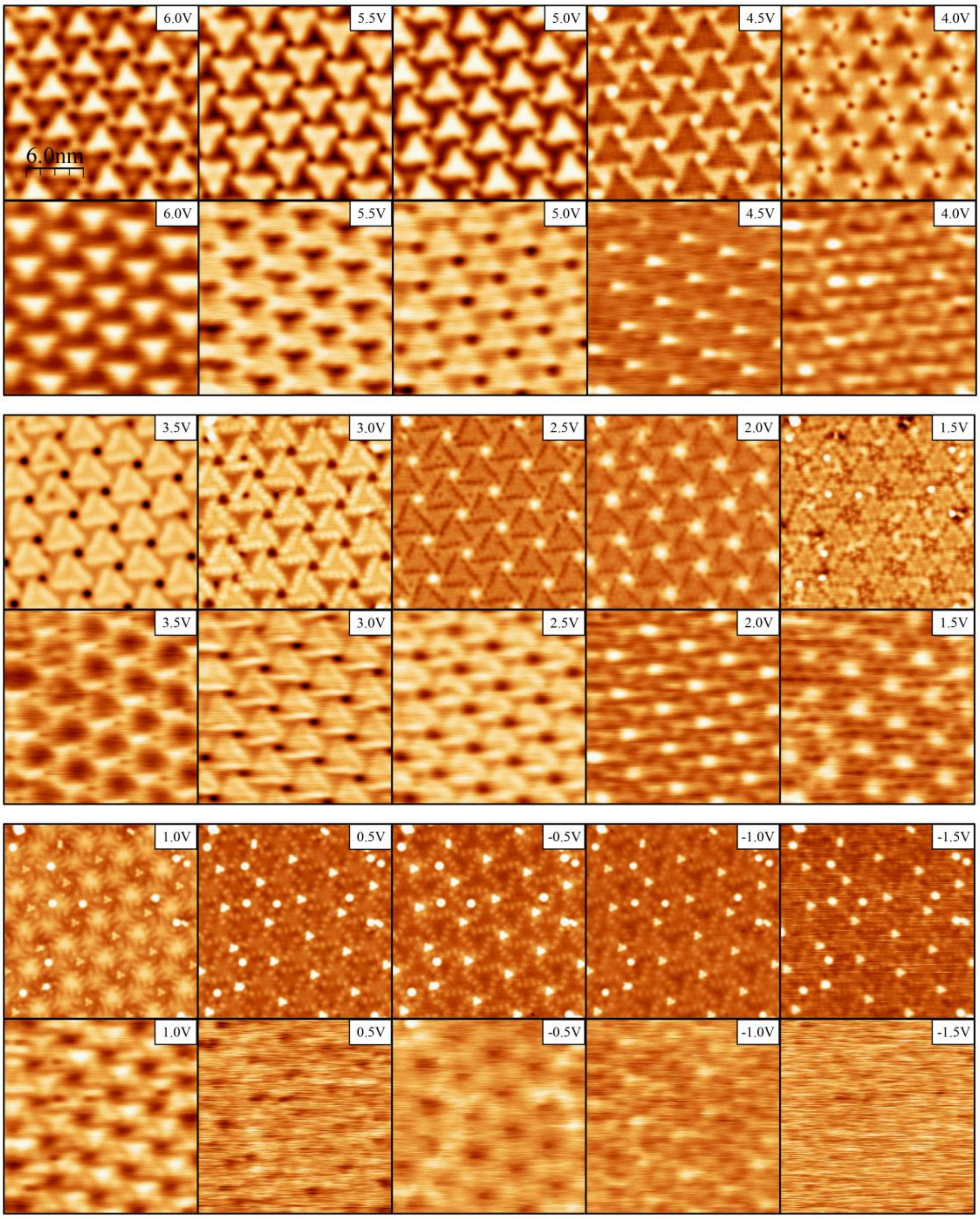

Supplementary Fig. 18 Series topographic images (20×20 nm^2) and corresponding d*I*/d*V* mappings of ultraflat chiral BlueP, the upper columns are the topographic images, and the bottom columns are the corresponding d*I*/d*V* mappings.

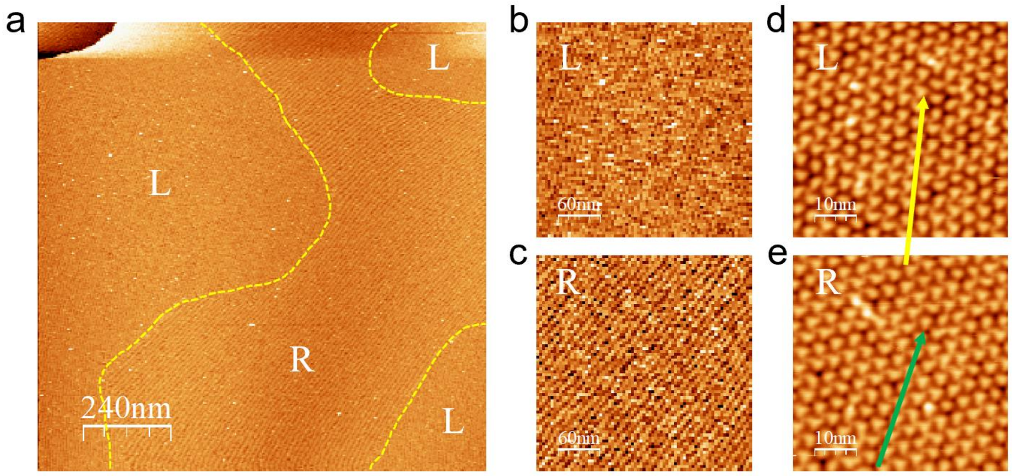

Supplementary Fig. 19 **a**, Large-area STM topography image (1.2 × 1.2 μm^2, V_S = 5.2 V, I_t = 200 pA), highlighting two chiral domains as marked as R and L. The single ultraflat chiral BlueP sheet can be up to μm in size. **b**,**c**, The L and R areas cropped directly from **a**, showing a moiré pattern in the R area. **d**,**e**, Enlarged STM images (30 × 30 nm^2, V_S = 5.2 V, I_t = 200 pA) of the L (left-handed chirality) and R (right-handed chirality) areas. In **(a)**, the image possesses 300 pixels per line, with a resolution of 4 nm/pixel, which is close to the period of chiral superstructures (~ 4.7 nm). Therefore, along a certain scanning direction, due to the different chiral superstructures having different vector basis directions, the lower resolution results in insufficient sampling points for the height information of the superstructures to form a moiré pattern. Therefore, in **(a)**, we can distinguish different chiral regions by the different moiré patterns.

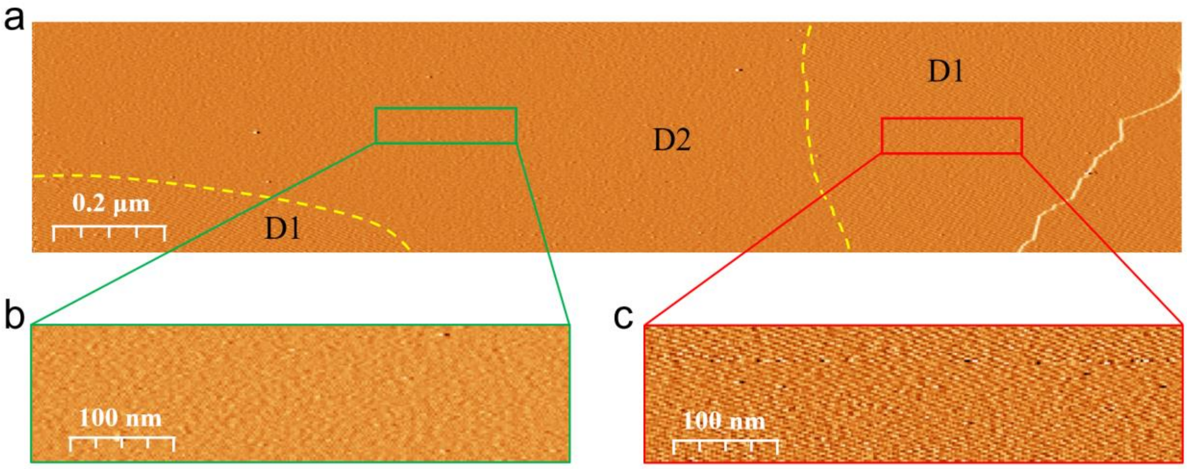

Supplementary Fig. 20 **a**, The derivative STM image corresponding to Figure 1d, showing two types of domain, D1 domain with an obvious period structure and D2 domain with a relatively flat morphology. **b**, The enlarged derivative STM image in the D2 domain. **c**, The enlarged derivative STM image in the D1 domain.

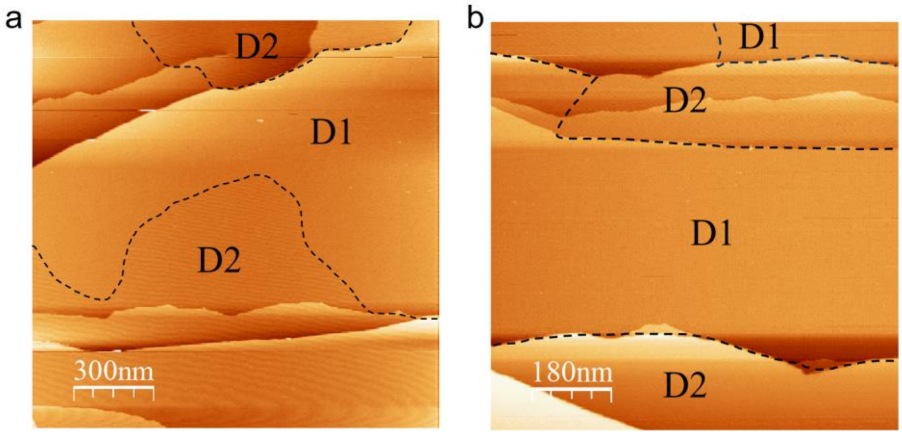

Supplementary Fig. 21 Large-area STM images (78K) of ultraflat chiral BlueP covered on Cu(111), with the different chiral area showing different morphology as marked in image. The ratio of two domains is close to 1:1.

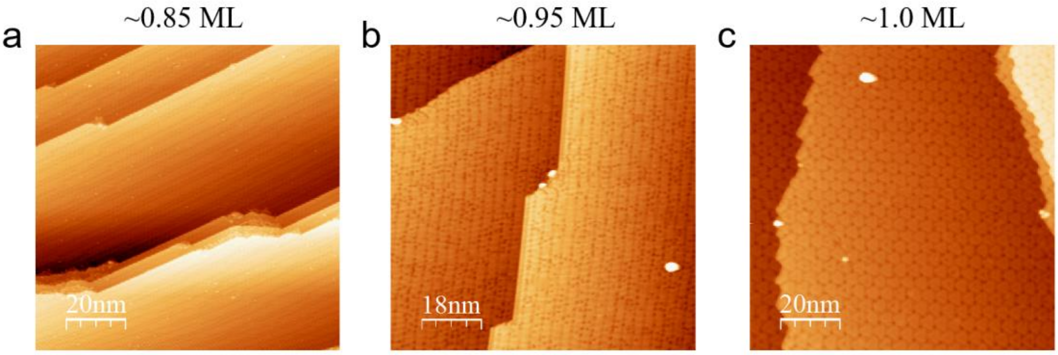

Supplementary Fig. 22 STM topographic images after growth of ~0.85 ML, 0.9 ML, and ~1ML phosphorous on Cu(111), in which the substrate is kept at ~260°C (the growth temperature used in the work of Kaddar et al.) and uses InP as the precursor.

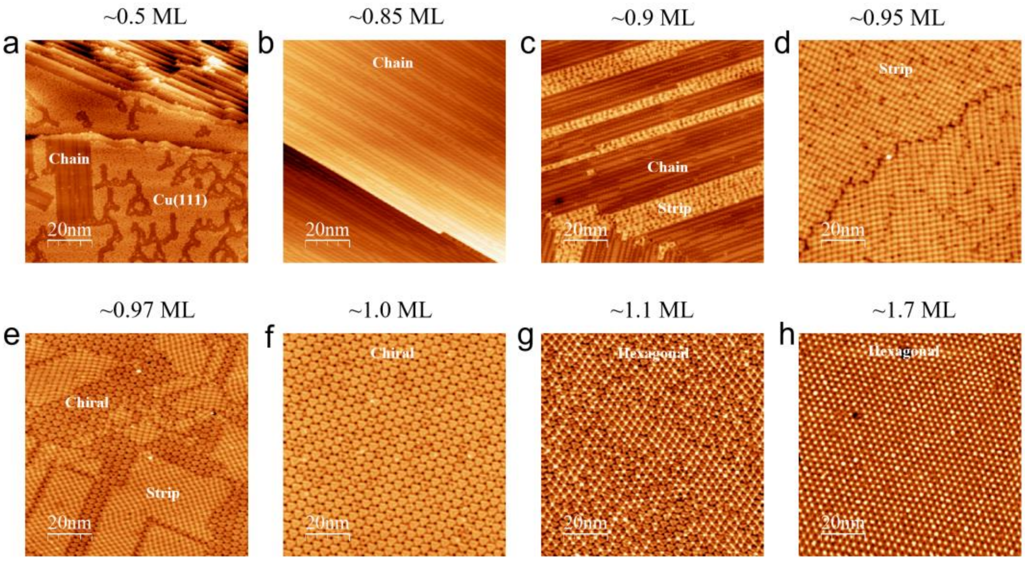

Supplementary Fig. 23 STM topographic images (78K) after growth of ~0.5 ML, ~0.85 ML, ~0.9 ML, ~0.95 ML, ~0.97 ML, ~ 1.0 ML, ~1.1 ML, and ~1.7 ML phosphorene on Cu(111) at ~200°C.

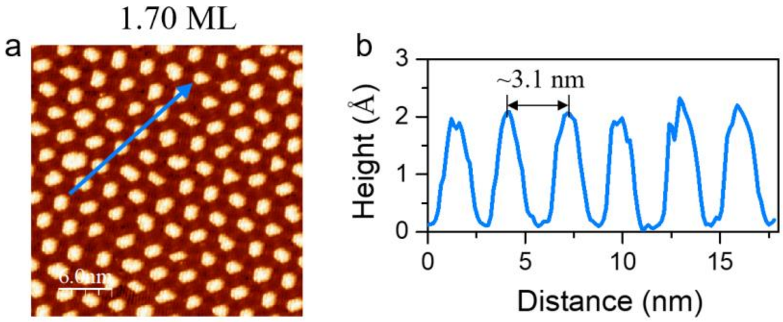

Supplementary Fig. 24 a, STM image of the phosphorene sheet at the coverage of 1.7 ML, highlighting the formation of the self-assembled P nanodots on its top. **b**, Line-profile along the blue line as indicated in the image, showing that the apparent height of the self-assembled P nanodots is ~2 Å, and the period of the P nanodots is ~3.1 nm.

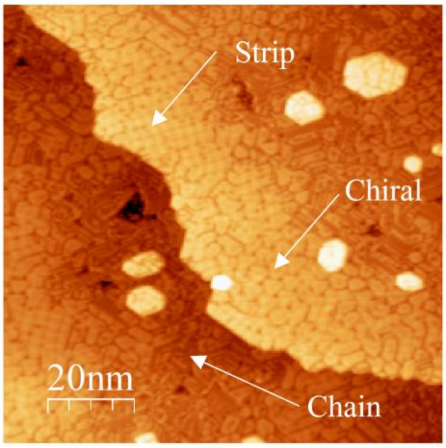

Supplementary Fig. 25 STM topographic image (78K) after depositing 0.8 ML phosphorous at ~180 °C, showing the coexistence of multiphases of phosphene, including the chain, strip, and chiral phases.

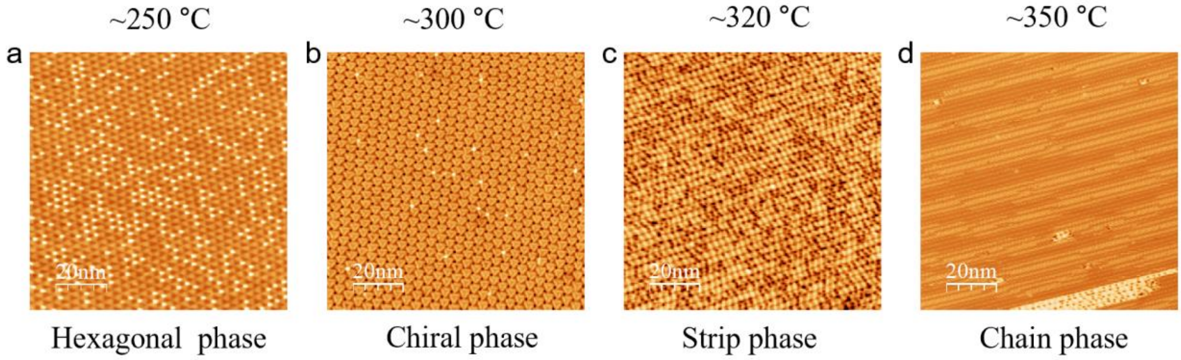

Supplementary Fig. 26 Stability of different phosphorene structures under annealing. **a-d**, STM images of the hexagonal, chiral, strip, and chain phases annealed at 250, 300, 320, and 350 °C, respectively, without the desorption of phosphorus atoms for each structure, signifying their stability.

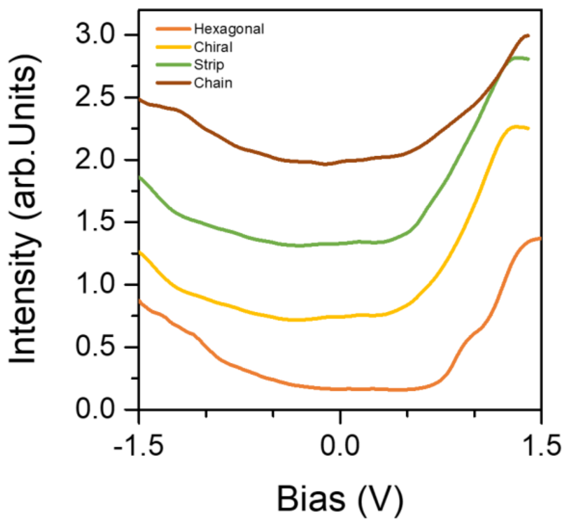

Supplementary Fig. 27 Large-energy scale STS taken on different phosphorene structures, including the chain, strip, chiral, and hexagonal phases, showing a metallic character for all the phases.

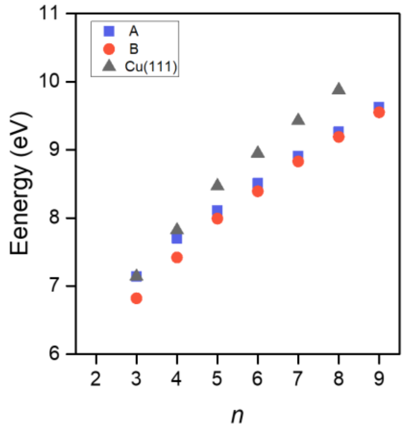

Supplementary Fig. 28 Extracted image-potential state peak positions from Figure 4d, which plot the V_n of the *n*th IPS with respect to *n*.

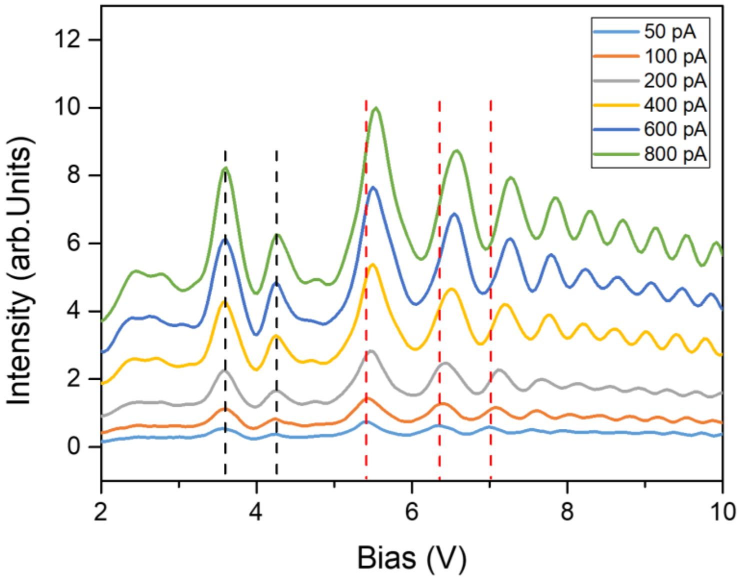

Supplementary Fig. 29 Field-emission resonance spectra on ultraflat chiral BlueP at different tunneling gap. According to equation 1, the V_n is proportional to E. Therefore, the image potential states would shift to higher energy with large electric filed or large tunneling currents, as indicated by red dashed lines. Meanwhile, the other electronic states are not expected to dependence the electric filed, as indicated by black lines. The feature marked by black lines can be ascribed to the intrinsic electronic states of ultraflat chiral BlueP and the interface states formed between chiral Blue ultraflat P and the Cu(111) substrate [4, 16].

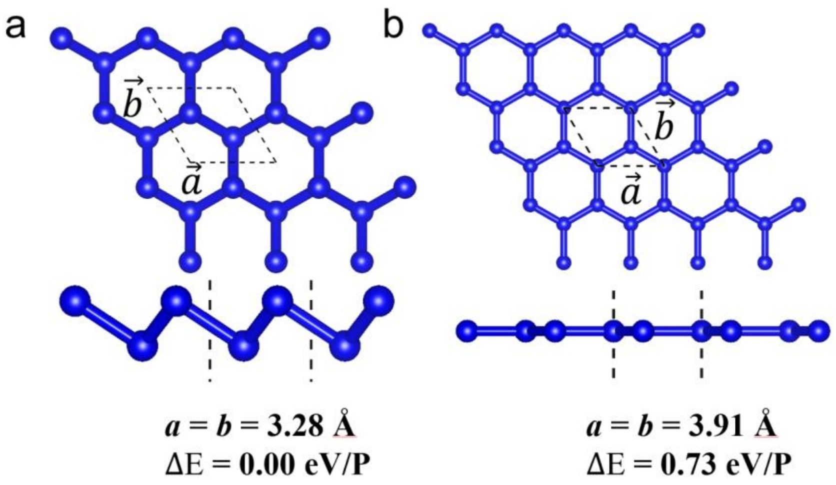

Supplementary Fig. 30 Optimized geometric structure of **(a)** buckled and **(b)** ultraflat BlueP. The ultraflat BlueP monolayer without substrate is energetically meta stable.

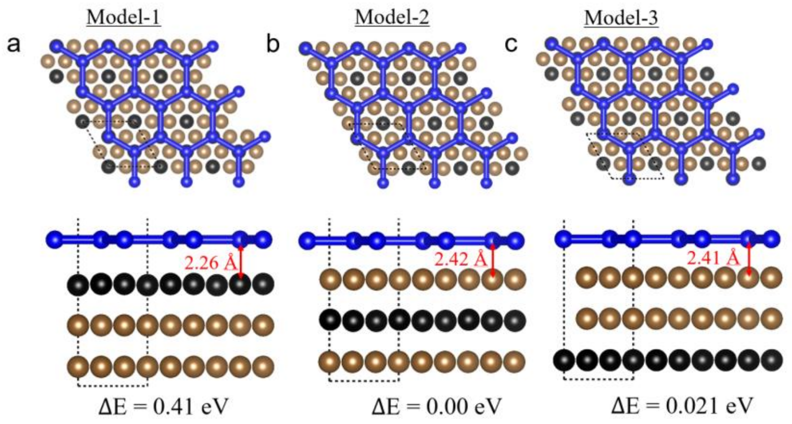

Supplementary Fig. 31 DFT calculations for three different models of ultraflat BlueP on Cu(111) substrate, with the corresponding unit cells indicated by the dashed-line rhombus. Blue and brown balls represent P and Cu atoms, respectively. As highlighted the atoms, the structure models with P atoms of the ultraflat BlueP on the **(a)** top sites **(b)** middle sites and **(c)** bottom sites of Cu(111) substrate, respectively. The Model-2 is the most stable configuration.

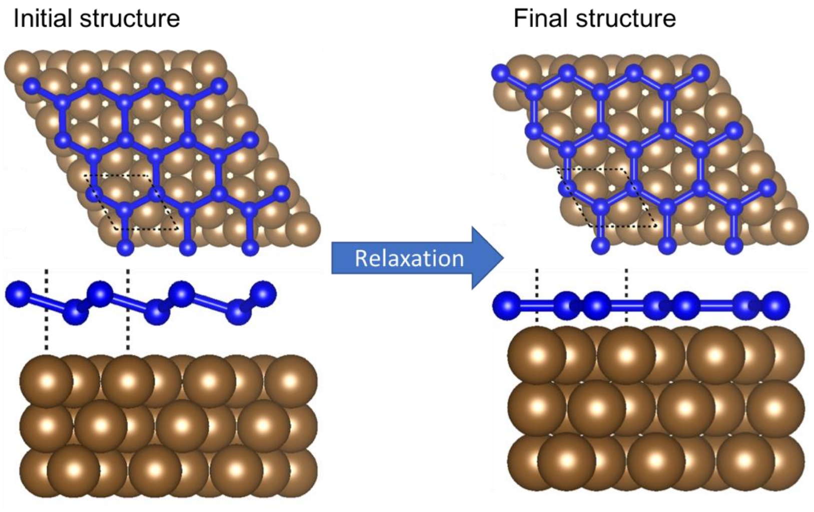

Supplementary Fig. 32 Structure evaluation of ultraflat BlueP on Cu(111). The buckled BlueP (1×1) is modelled on Cu(111) with the surface of 4.18 Å (experimental measured value). Due to large value of lattice mismatch (δ), the buckled BlueP become flat after structure optimization. The calculated E_b of ultraflat BlueP is found as 0.56 eV/P atom, suggesting a strong interaction with the substrate.

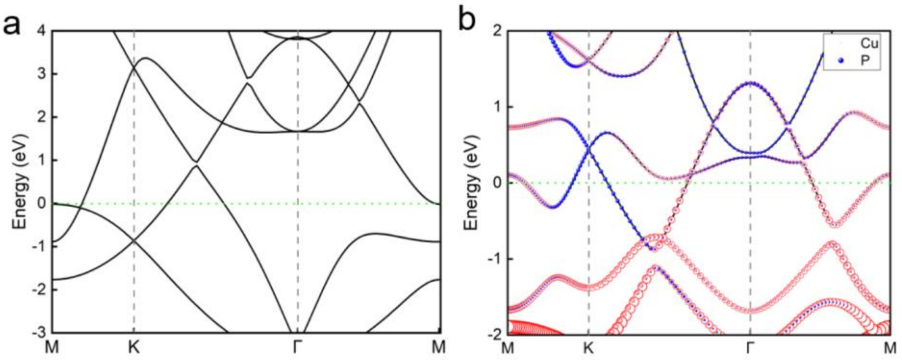

Supplementary Fig. 33 Electronic band structures of the ultraflat BlueP **(a)** in vacuum and **(b)** on Cu(111) surface.

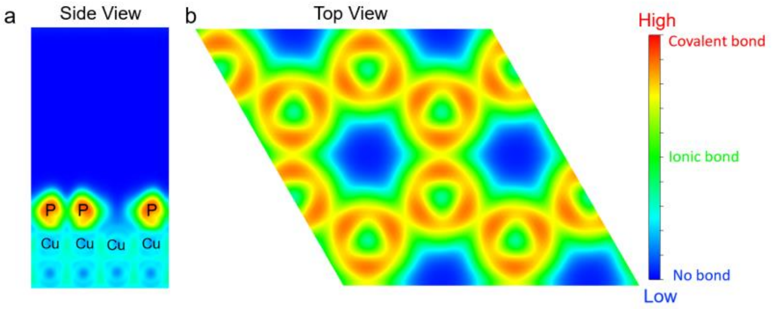

Supplementary Fig. 34 Cross-sectional ELF showing high localization of the electrons in P−P pairs and weak P−Cu interaction.

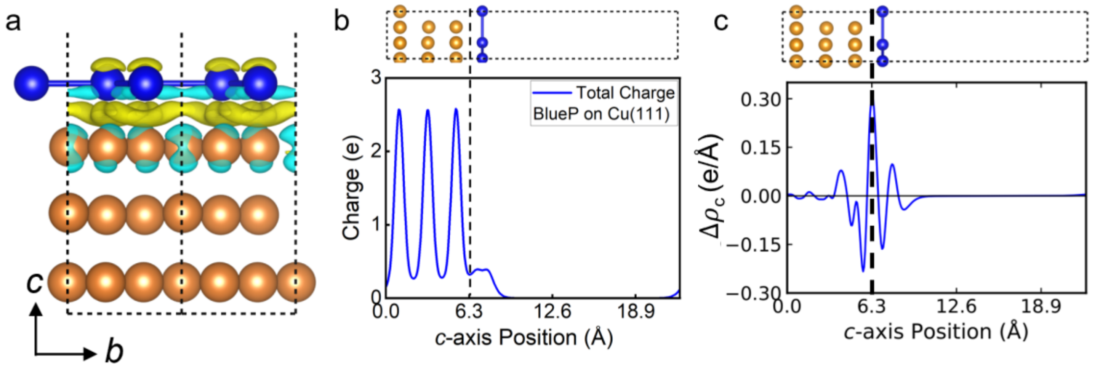

Supplementary Fig. 35 a, Charge density difference of the ultra-flat BlueP on Cu (111). The cyan (yellow) zone gains (losses) charge with isosurface value of ±0.004|e|/bohr^3. **b**,**c**, Total charge and charge density difference distributions for the ultraflat BlueP on Cu(111).

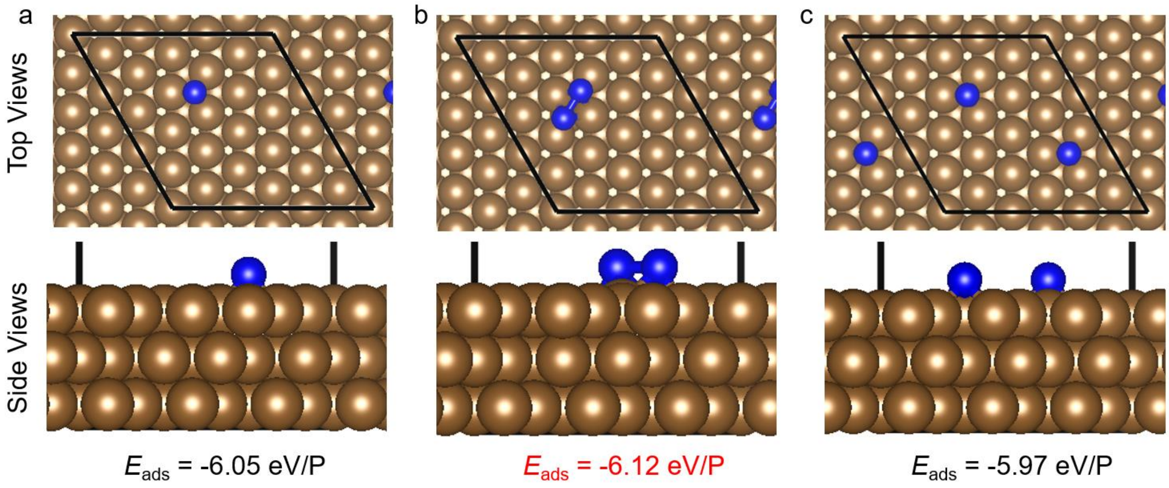

Supplementary Fig. 36 Top (upper panel) and side (lower panel) views of the most stable configurations of (**a**) a P monomer, (**b**) a P-P dimer, and (**c**) two separated P adatoms on the Cu(111) surface.

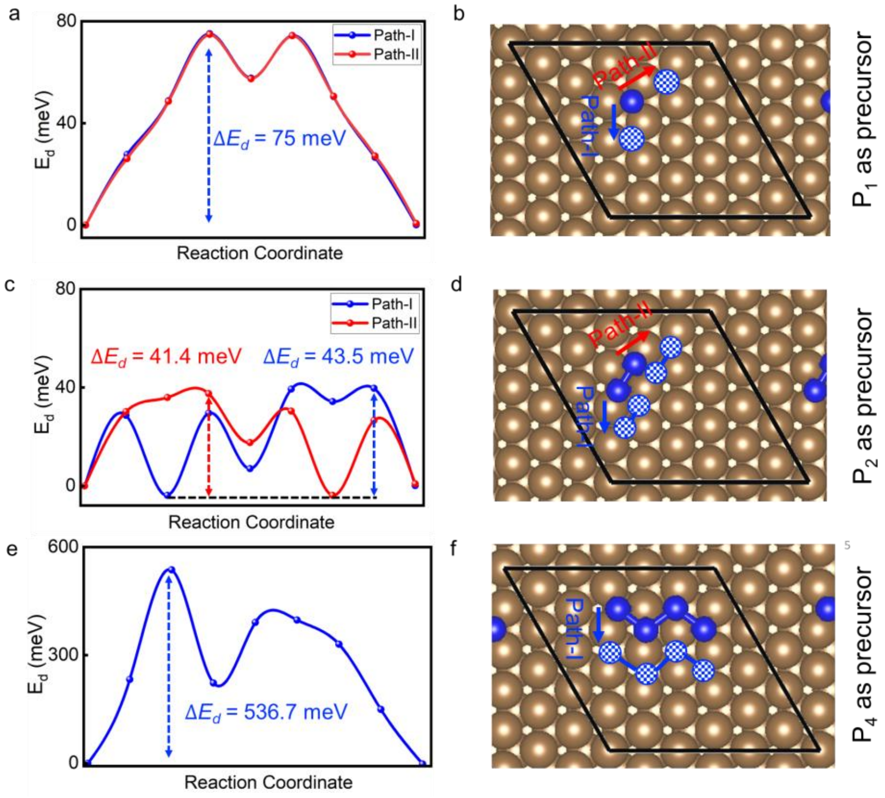

Supplementary Fig. 37 a, Diffusion pathways of a P monomer on Cu(111), with corresponding energy profiles presented in (**b**). **c**-**d** and **e**-**f** depict the same information as (**a**) and (**b**), respectively, but for the cases of an adsorbed P-P dimer and P_4. The solid and dotted blue balls represent the initial and final positions of the P adatoms, respectively.

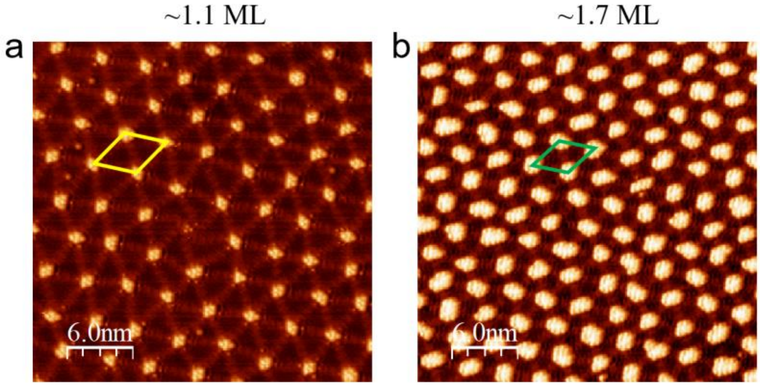

Supplementary Fig. 38 Enlarged STM images of 1.1 ML and 1.7 ML phosphorene on Cu(111). The yellow and green diamonds mark the unit cell of hexagonal superstructures. For 1.1 ML phosphorene, only several P atoms are located on the nodes, but for 1.7 ML phosphorene, there are P clusters located on the nodes (the same P nanodots structure as the recent work[2] of Kaddar et al.).

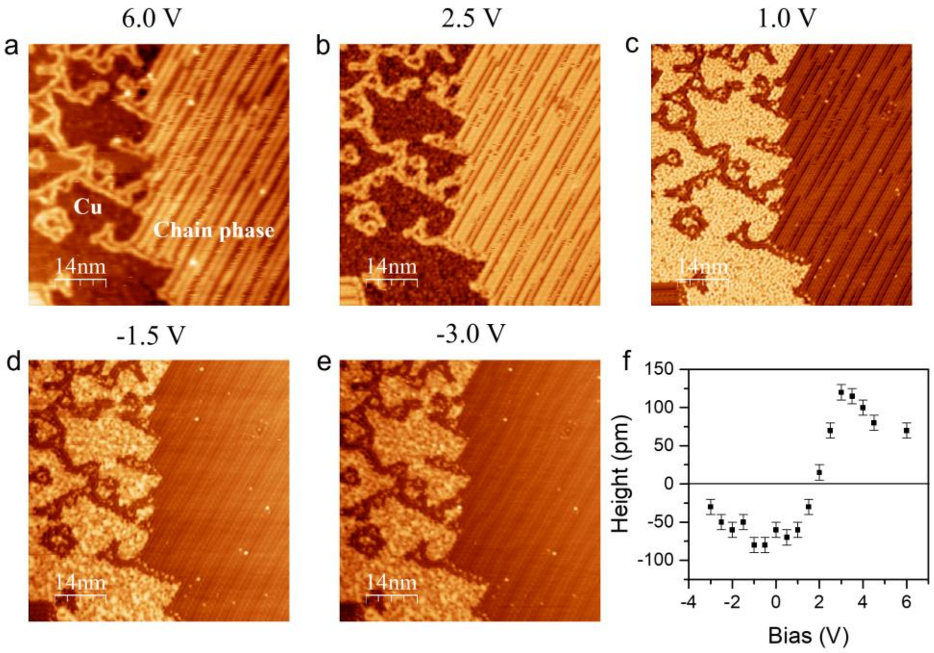

Supplementary Fig. 39 **a-e**, A series of STM images of the same area with different bias voltages for the chain phase of phosphorene on Cu(111). **f**, The extracted step height of phosphorene islands from different tip bias STM images. The phosphorene islands are higher than the surrounding Cu(111) terrace (**a-b**) with a bias voltage larger than 2.0 V. While below 2.0 V, the phosphorene islands appear lower than the Cu(111) terrace (**c-e**). The bias-dependent height demonstrates the strong influence of the LDOS on the height measurement by STM.

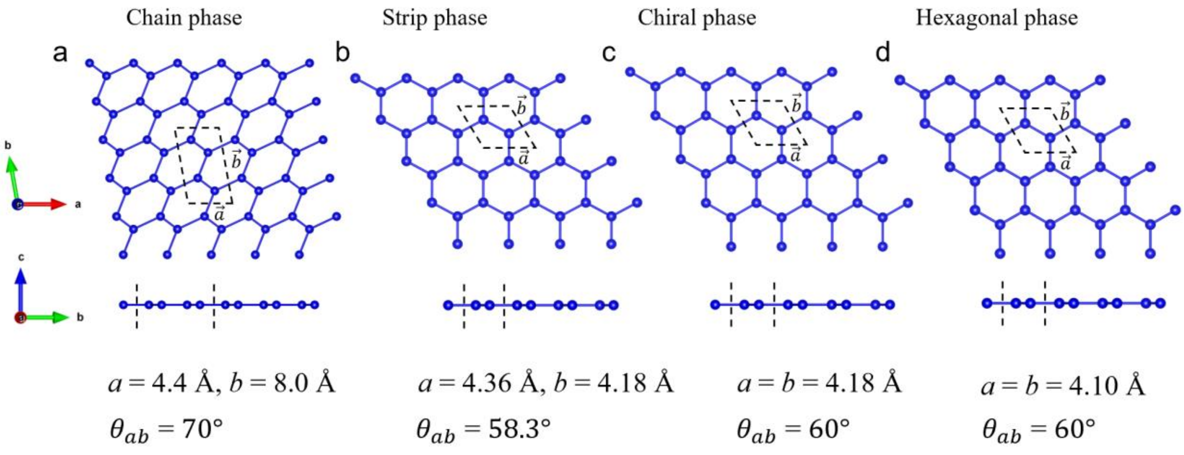

Supplementary Fig. 40 Proposed atomic structures for chain, strip, chiral, and hexagonal phases of phosphorene, respectively, showing an ultraflat pseudo-honeycomb in (**a**-**b**) or honeycomb structure (**c**-**d**).

Supplementary Table. 1 Coverages of different phosphorene phases calculated by proposed atomic structures and obtained from the experiments.

	Chain phase	Strip phase	Chiral phase	Hexagonal phase
Calculated coverage	0.91 ML	0.97 ML	1.0 ML	1.04 ML
Experimental coverage	0.85 ML	0.95 ML	1.0 ML	1.1 ML

Supplementary References

1. Kim, C. J., Sanchez-Castillo, A., Ziegler, Z., Ogawa, Y., Noguez, C. & Park, J. Chiral atomically thin films. *Nat. Nanotechnol.* **11**, 520-524 (2016).

2. Kaddar, Y. *et al.* Dirac Fermions in Blue Phosphorene Monolayer. *Adv. Funct. Mater.* **33**, 2213664 (2023).

3. Hÿtch, M. J., Snoeck, E. & Kilaas, R. Quantitative measurement of displacement and strain fields from HREM micrographs. *Ultramicroscopy* **74**, 131-146 (1998).

4. Liu, X., Li, Q., Ruan, Q., Rahn, M. S., Yakobson, B. I. & Hersam, M. C. Borophene synthesis beyond the single-atomic-layer limit. *Nat. Mater.* **21**, 35–40 (2021).

5. Kolesnychenko, O. Y., Kolesnichenko, Y. A., Shklyarevskii, O. I. & van Kempen, H. Field-emission resonance measurements with mechanically controlled break junctions. *Phys. B* **291**, 246-255 (2000).

6. Feng, B. *et al.* Experimental realization of two-dimensional boron sheets. *Nat. Chem.* **8**, 563-568 (2016).

7. Zhang, J. L. *et al.* Epitaxial growth of single Layer blue phosphorus: a new phase of two-dimensional phosphorus. *Nano Lett.* **16**, 4903-4908 (2016).

8. Putyato, M. A. *et al.* A valved cracking phosphorus beam source using InP thermal decomposition and its application to MBE growth. *Semicond. Sci. Technol.* **24**, 055014 (2009).

9. Zhang, Z. & Lagally, M. G. Atomistic Processes in the Early Stages of Thin-Film Growth. *Science* **276**, 377-383 (1997).

10. Chen, H., Zhu, W. & Zhang, Z. Contrasting behavior of carbon nucleation in the initial stages of graphene epitaxial growth on stepped metal surfaces. *Phys. Rev. Lett.* **104**, 186101 (2010).

11. Wu, P., Zhang, Y., Cui, P., Li, Z., Yang, J. & Zhang, Z. Carbon dimers as the dominant feeding species in epitaxial growth and morphological phase transition of graphene on different Cu substrates. *Phys. Rev. Lett.* **114**, 216102 (2015).

12. Li, X. *et al.* Large-area synthesis of high-quality and uniform graphene films on copper foils. *Science* **324**, 1312-1314 (2009).

13. Li, X., Cai, W., Colombo, L. & Ruoff, R. S. Evolution of graphene growth on Ni and Cu by carbon isotope labeling. *Nano Lett* **9**, 4268-4272 (2009).

14. Loginova, E., Bartelt, N. C., Feibelman, P. J. & McCarty, K. F. Factors influencing graphene growth on metal surfaces. *New J. Phys.* **11**, 063046 (2009).

15. Loginova, E., Bartelt, N. C., Feibelman, P. J. & McCarty, K. F. Evidence for graphene growth by C cluster

attachment. *New J. Phys.* **10**, 093026 (2008).

16. Ruggiero, C. D., Choi, T. & Gupta, J. A. Tunneling spectroscopy of ultrathin insulating films: CuN on Cu(100). *Appl. Phys. Lett.* **91**, 253106 (2007).
